# Acute Fibrinous and Organizing Pneumonia and Undifferentiated Connective Tissue Disease: A Case Report

**DOI:** 10.1155/2012/549298

**Published:** 2012-04-04

**Authors:** Valéria Valim, Roberta Hora Rocha, Roberta Barcelos Couto, Thaysa Simões Paixão, Érica Vieira Serrano

**Affiliations:** Department of Internal Medicine, Universidade Federal do Espirito Santo, Marechal Campos Avenue 1355, 29040-715 Vitoria, ES, Brazil

## Abstract

Acute fibrinous and organizing pneumonia (AFOP), recently described, is a histologic pattern characterized by the presence of fibrin “balls” within alveolar spaces. The term undifferentiated connective tissue disease (UCTD) is used to identify autoimmune systemic diseases that do not fulfill the criteria to be classified as a definitive connective tissue disease. The AFOP has never been reported in association with UCTD. The present reported case is a 39-year-old Caucasian, female with dry cough and progressive dyspnea. Eight months later, she was diagnosed with “organizing pneumonia” based on clinical history and radiologic images. She manifested Raynaud's Phenomenon, sicca syndrome, boot and gloves neuropathic pain, and previous hypothyroidism. Antinuclear antibody, rheumatoid factor, and specific autoantibodies were negative. Salivary gland biopsy and electroneuromyiography were normal. The capillaroscopy showed a “scleroderma” pattern with capillary deletion and ectasia. She experienced clinical and radiologic worsening. Despite being submitted to cyclophosphamide pulse, she developed hemorrhage and then died. Thoracotomy pulmonary specimen showed histological pattern of AFOP. This paper shows a rare association of AFOP with UCTD.

## 1. Introduction

Acute fibrinous and organizing pneumonia (AFOP) is an unusual type of lung injury characterized by a typical histological pattern recently described whose main features are the presence of intra-alveolar fibrin in the form of “balls” and organizing pneumonia with patchy distribution, which does not meet the classic histologic criteria for bronchiolitis obliterans with organizing pneumonia (BOOP), eosinophilic pneumonia (EP), or diffuse alveolar damage (DAD) [1–3].

This entity described in 2002 by Beasley et al. presents clinically as a lung injury. There seem to be two distinct patterns of disease progression and outcome: an acute, with fulminant course and rapid progression to death, and subacute, with chances of survival and recovery [1].

Acute fibrinous and organizing pneumonia (AFOP) may be idiopathic in nature or associated with many factors, such as autoimmune rheumatic diseases, occupational exposure (exotic animals, coal and tungsten mines, and hairspray [1, 4]), altered immune status (long-term steroids use, poorly controlled diabetes, alcoholism, lymphoma, acute myeloid leukemia and allogeneic hematopoietic stem cell transplantation, HIV infection, and *Pneumocystis jiroveci* pneumonia [1, 5, 6]), infectious causes (*Haemophilus influenzae*, *Acinetobacter baumannii,* and coronavirus [1, 3]), and exposure to drugs (abacavir, decitabine, and amiodarone) [1, 7–9].

The term undifferentiated connective tissue disease (UCTD) is used to identify autoimmune systemic diseases that do not fulfill the criteria to be classified as a definitive connective tissue disease (CTD) such as systemic lupus erythematosus, Sjögren's syndrome, systemic sclerosis, mixed connective tissue disease, rheumatoid arthritis, systemic vasculitis, and polymyositis/dermatomyositis. Some studies observed that this condition could progress into some of these diseases. The onset of UCTD is similar to most CTDs, peaking in the middle years of life [10, 11].

The natural history of these rare entities is variable: a high percentage of patients with UCTD maintain an undifferentiated clinical course and do not evolve to a distinct CTD, whereas some patients can evolve over time [11].

Lung involvement as the first clinical manifestation of UCTD is rarely reported and usually appears as a complication in established UCTD [11].

There are only six cases of AFOP associated with rheumatic disease described.

There is no previously published case of AFOP and UCTD.

## 2. Case Report

A 39-year-old female Caucasian psychologist sought medical attention complaining of cough. Chest radiography showed changes that were attributed to pneumonia, treated with cefuroxime at the time. After eight months, progressive dyspnea was associated.

**Figure 1:**
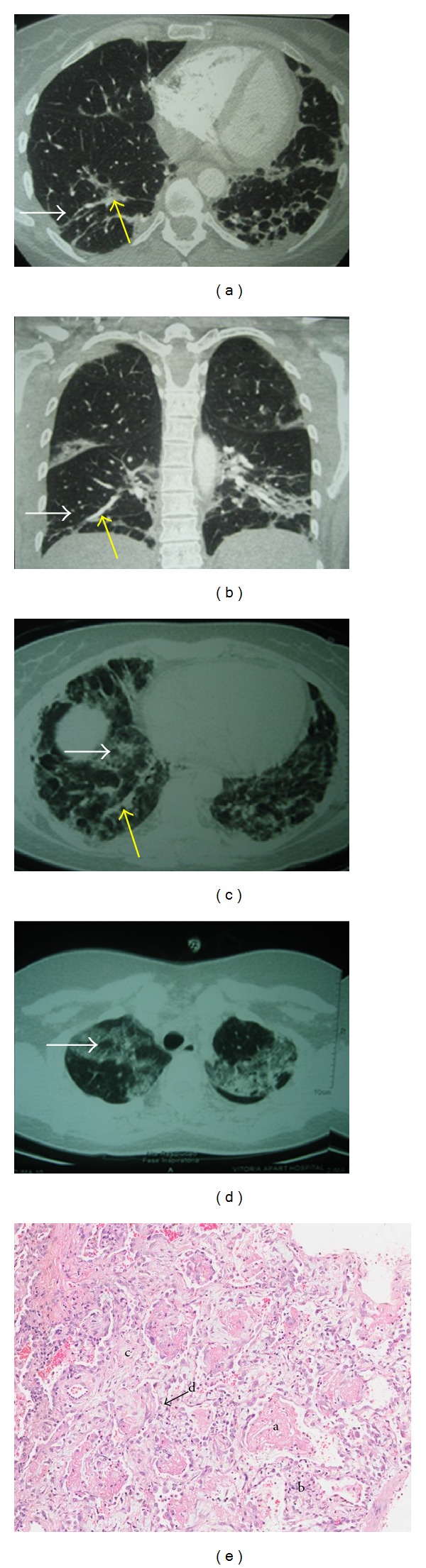
(a) and (b) CT findings of “focal areas of lung parenchymal infiltrates with ground-glass opacities, associated (white arrow) with septal thickening (yellow arrow), with peripheral distribution.” (c) and (d) CT findings of interstitial infiltrate, with diffuse ground glass opacity with foci of parenchymal densification (yellow arrow) and some air bronchograms (white arrow). (e) Histhopatological examination showing (1) intra-alveolar accumulation of fibrin in the form of solid blocks; (2) acute inflammatory infiltrate in the interstitium; (3) alveolar septal edema and congestion; (4) *xantomatososum* macrophages accumulation.

Initial diagnosis by pulmonologist was “organizing pneumonia,” based on clinical history and computed tomographic (CT) findings, described as “focal areas of lung parenchymal infiltrates with ground-glass opacities, associated with septal thickening, with peripheral distribution, predominating in the lower lobes” (Figures 1(a) and 1(b)). Transbronchial biopsy was performed, which showed “scant fragments of bronchial wall and adjacent alveolar collapse exhibiting a minimum focus of interstitial inflammatory infiltrate.” It was prescribed treatment with prednisone 20 mg/day.

After twelve months of evolution since the onset of symptoms, she went to a rheumatologist for differential diagnosis evaluation. On this occasion, the pulmonologist had already searched and excluded tuberculosis and she had dyspnea on minimal exertion. Diffuse pain in arms and legs, burning in the fingertips, fatigue, sleep disorder, Raynaud's phenomenon (RP), xerostomia, and xerophthalmia were present at the symptomatology interrogation and physical examination. She had positive personal and family history of Hashimoto's thyroiditis. On physical examination there was no skin thickening or arthritis, oroscopy was normal, blood pressure was 90 × 60 mmHg, and heart frequency was 96 beats per minute. Upon respiratory system examination basal crackles bilaterally and 20 breaths per minute were observed. There were no changes in cardiovascular and abdominal examination.

Tests were requested for the investigation of rheumatic diseases including systemic sclerosis, Sjögren's syndrome, and vasculitis. On this occasion, the prednisone dose was increased to 1 mg/kg/day.

Inflammatory activity tests and serologic and antibodies tests are demonstrated in Table 2.

In the evaluation of lung function a pattern compatible with incipient restrictive ventilatory disorder was observed. As evidence of inflammatory activity, CRP was always negative and ESR was increased. Autoantibodies were negative (antinuclear antibodies-ANA, rheumatoid factor, anti-SCL 70, anti-Jo-1, anti-RNP, anti-SS-A, anti-SS-B, and c-ANCA), except a 1 : 20 p-ANCA reagent. The patient presented a normocytic normochromic anemia, and the hemoglobin value ranged from 9.8 to 13.3 mg/dL and the hematocrit range was 30.3 to 41.7%, probably due to the underlying disease. In two occasions, the patient presented lymphopenia that could indicate disease activity or could be related to steroid therapy. Chlamydia pneumonia IgG serology was reagent 1 : 512 and IgM was negative. Mycoplasma pneumonia serology was negative. The exams allowed excluding Sjögren's syndrome (negative anti-SS-A and anti-SS-B and normal salivary biopsy). Renal, liver, and hormonal functions were normal.

**Figure 2:**
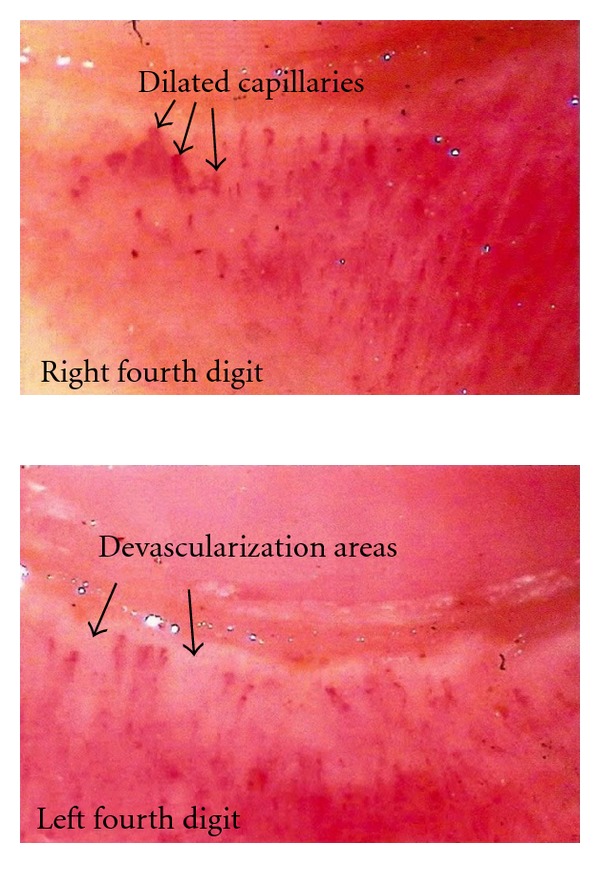
Panoramic nailfold capillaroscopy of the left and right fourth digits showing a scleroderma standard microangiopathy: dilated capillaries and devascularization areas.

A panoramic nailfold capillaroscopy showed the presence of moderate capillary ectasia and mild devascularization of focal distribution, described as “well-defined scleroderma (SD) standard microangiopathy, consistent with systemic sclerosis, dermatomyositis or overlap syndrome with scleroderma component” (Figure 2). 

Given the results of capillaroscopy and the presence of RP she was diagnosed with systemic sclerosis in the early stages. Then it was proposed to start cyclophosphamide pulse 1 g.

However, after three weeks of diagnosis, the patient developed high fever (39 degrees) and dyspnea at rest, requiring hospitalization. On admission, her respiratory frequency was 40 incursions per minute, the arterial oxygen tension was 48.4 mmHg, and oxygen saturation was 88.5% and she had basal crackles bilaterally. On the third day she developed respiratory failure, requiring ventilatory support and intensive care. The chest CT showed interstitial infiltrate, with diffuse ground-glass opacity in both lungs, associated with foci of parenchymal densification, predominantly peripheral, some air bronchograms and mild pleural thickening marginally, more evident in right lung basis and apex. Small calcified nodules of residual aspect, scattered in both lungs, without pleural effusion (Figures 1(c) and 1(d)), were observed. An echocardiogram was done to exclude pulmonary hypertension. The pulmonary arterial pressure was 31 mmHg.

Hemoculture and urine culture were negative. Virus B and C serologies were negative. The CRP increased from 42 to 147 mg/dL.

At that moment the pulmonologist considered three possible diagnosis: nonspecific interstitial pneumonia, pneumonia caused by *Pneumocystis carinii,* or pneumonia caused by virus (CMV, adenovirus, herpes 1 and 2).

On the fourth day, she underwent an open biopsy (microthoracotomy), which consisted of pulmonary segmentectomy of right middle and lower lobes. Some part of the material was sent to investigate fungus, mycobacteria, and general culture, all of them presented negative results. The biopsy revealed that there were no infectious process and no granulomas. Histopathological examination showed “a parenchymal diffuse involvement, intra-alveolar fibrin accumulation in the form of solid blocks, pneumocytes hyperplasia, interstitial mild acute inflammatory infiltrate, alveolar septal edema and congestion, intra-alveolar fibroblastic Masson bodies and *xantomatososum * accumulation of macrophages, a typical pattern of acute Fibrinous and Organizing Pneumonia (AFOP)” (Figure 1(e)).

After excluding any infection etiology including bacteria, virus, mycobacteria, and fungus, she received pulse therapy with cyclophosphamide 1 g and methylprednisolone 1 g for three days. Despite treatment, the patient developed respiratory failure by pulmonary hemorrhage.

## 3. Discussion

We reported the case of a female patient of 39 years with diagnosis of undifferentiated connective tissue disease (UCTD) and manifestations suggestive of systemic sclerosis (SSc) as Raynaud's phenomenon (RP) and capillaroscopic pattern SD associated with acute fibrinous and organizing pneumonia (AFOP).

There were some difficulties to complete the diagnosis in relation to both pulmonary and systemic aspects.

A detailed assessment by a rheumatologist specialist noted the presence of RP, which is a symptom guide to SSc diagnosis [15, 16]. Raynaud's phenomenon is one of the most common symptoms in SSc (90–95%) but also can be find in other rheumatic diseases like lupus, rheumatoid arthritis, Sjögren's syndrome, myositis, and overlap diseases. It often precedes rheumatic disease manifestations by a variable period of time [15, 17]. At the present time capillaroscopy is recommended as mandatory exam to investigate RP for the diagnosis of early SSc [16, 18, 19]. In patients with RP, the presence of capillaroscopy abnormalities that characterize the scleroderma pattern indicates a high specificity and a positive predictive value for the diagnosis of SSc and related disorders, more than a positive antinuclear antibodies test [16].

The manifestations strongly suggested the SSc, but it was not possible to give the diagnosis based on the American College of Rheumatology criteria [20]. Also the patient did not have skin or characteristic pulmonary manifestation. Then, we concluded with the diagnosis of UCTD.

Acute fibrinous and organizing pneumonia (AFOP) is a histological pattern characterized by intra-alveolar fibrin accumulation associated with organizing pneumonia consisting of intraluminal loose connective tissue within the alveolar bronchioles and ducts associated with the fibrin [1, 2]. In this case, the histopathological study of the patient showed a lung parenchymal diffuse involvement, intra-alveolar fibrin in the form of solid blocks, pneumocytes hyperplasia, mild acute inflammatory infiltrate in the interstitium, alveolar septal edema and congestion and intra-alveolar fibroblastic Masson bodies, also reported by Beasley et al. [1].

Beasley et al. noted that the period between the onset of symptoms and the biopsy performance did not exceed two months, and, among patients who died, the average number of days between onset of symptoms and death was 29 days [1]. In this patient, 15 months have passed from the onset of initial symptoms until the biopsy and death. The need for mechanical ventilation (MV) was the only significant parameter that correlated with prognosis in cases studied by Beasley et al., and all patients who required mechanical ventilation died [1]. The same was observed in the patient of this case.

Steroids and antibiotics, associated or not, diuretics and mechanical ventilation were used in the treatment of AFOP by Beasley et al., and therapeutic modality used did not correlate with the outcome background [1]. It was not found yet an ideal treatment for AFOP, but there are reports in the literature of successful treatments with mycophenolate mofetil and methylprednisolone [21] and prednisone and cyclophosphamide [22]. The patient in this study received cyclophosphamide and methylprednisolone pulse therapy in the intensive critical unit but progressed to respiratory failure by pulmonary hemorrhage.

We believe that early therapy institution could have resulted in a more favorable outcome in this case, although further studies are still needed on this new entity to support this understanding.

Clinically, the diagnosis of AFOP is undistinguishable from the others lung injuries diagnosis. Adding the radiologic aspects there is a range of differential diagnoses as atypical pneumonia, pulmonary edema, and interstitial pneumoniae [1]. In the presented current case, initially pneumonia was diagnosed. Then, according to the clinical and radiographic aspects she was diagnosed with organizing pneumonia (OP). However, the result of the histopathological examination showed, posteriorly, the AFOP standard. The diagnosis of AFOP is essentially histological. In the series studied by Beasley et al., cases of patients who had only transbronchial biopsy were excluded from the study [1] since, due to the localized nature of the injury, this kind of biopsy may fail on histologic diagnosis [1, 2]. Acute fibrinous and organizing pneumonia (AFOP) diagnosis should only be made on large biopsy specimen when it is quite certain that there are not otherwise diagnostic features of diffuse alveolar damage (DAD), eosinophilic pneumonia (EP) or OP, histological patterns that make differential diagnosis with AFOP [2]. No hyaline membranes were seen as in DAD, and despite the presence of eosinophils in some specimens, they were never predominant as in EP [22]. Although fibrin deposits have been described in both the DAD and OP patterns, they do not comprise a major component of the process [1].

Regarding the association with autoimmune rheumatic diseases and AFOP, Beasley et al. noted in their series an association with ankylosing spondylitis and polymyositis [1]. Association with rheumatic diseases as systemic lupus erythematosus and juvenile dermatomyositis was already described in literature [12–14] (Table 1).

There are no previous reports in the literature about the association of UCTD and AFOP and this is the first reported case. It is a rare association that may be increasingly reported in the literature as this new terminology is best known and most biopsies are early performed with proper technique.

## 4. Conclusion

In this paper it was presented a rare combination of acute fibrinous and organizing pneumonia (AFOP) with an undifferentiated connective tissue disease (UCTD).

## Figures and Tables

**Table 1:** Described cases of acute fibrinous organizing pneumonia, 2002–2011.

Rheumatologic disease	Age	Sex	Treatment	Time	Outcome evolution	Author, year
Ankylosing spondylitis	55	M	Corticoid	—	Survived	Beasley et al., 2002, [1]
Polymyositis	78	F	Corticoid	—	Death	Beasley et al., 2002, [1]
Fibromyalgia	58	F	Antibiotic	—	Survived	Beasley et al., 2002, [1]
Juvenile dermatomyositis	14	F	Azithromycin, immunoglobulin, cyclophosphamide, methylprednisolone, and cyclosporine	2 months	Death	Prahalad et al., 2005, [12]
Stigma of rheumatic disease	47	M	Prednisone	3 months	Survived	Balduin et al., 2007, [13]
SLE and APS	16	M	Prednisone, cyclophosphamide, anticoagulants	2,5 months	Survived	Hariri et al., 2010, [14]

SLE: systemic erythematosus lupus; APS: antiphospholipid syndrome.

**Table 2:** Inflammatory activity tests and serologic and autoantibodies tests.

	09/12/2007	10/30/2007	03/06/2008	07/02/2008
ESR (mm/h) RV: <20	67	54		55
CRP (mg/dL) RV: <0,5	<0,5	<0,5		<0,5
Total protein (g/dL) RV: 6–8		72		
ANA RV: nonreactive	Nonreactive	Nonreactive	Nonreactive	
RF (UI/mL) RV: <40	<40	<40	<40	
P-ANCA RV: negative			1 : 20	
Anti-TPO (UI/mL) RV: <35			842	
Anti-TBG (UI/mL) RV: <40			141	

Negative autoantibodies: anti-Scl70, anti-Jo1, c-ANCA, anti-RNP, anti-SS-A, anti-SS-B, anti-CCP, antinative DNA, and anticentromere.				

*Mycoplasma pneumoniae*, *Chlamydia pneumoniae*, HBV and HCV negative serologic tests.				

ESR: erythrocyte sedimentation rate; CRP: C-reactive protein; ANA: antinuclear antibodies; RF: rheumatoid factor; P-ANCA: perinuclear antineutrophil cytoplasmic antibodies; anti-TPO: antithyroid peroxidase; anti-TGB: antithyroxine binding globulin; anti-Scl 70: antitopoisomerase antibodies; anti-Jo 1: antisynthetase antibodies; c-ANCA: cytoplasmic antineutrophil cytoplasmic antibodies; anti-RNP: antiribonucleoproteins antitopoisomerase antibodies; anti-SS-A: anti-Sjogren's Syndrome antigen A; anti-SS-B: anti-Sjogren's syndrome antigen B; anti-CCP: anticitrulline containing peptide; HBV: hepatitis B virus; HCV: hepatitis C virus; RV: reference value.
